# Individuals at Risk of Exercise Addiction Have Higher Scores for Depression, ADHD, and Childhood Trauma

**DOI:** 10.3389/fspor.2021.761844

**Published:** 2022-01-26

**Authors:** Flora Colledge, Ursula Buchner, André Schmidt, Gerhard Wiesbeck, Undine Lang, Uwe Pühse, Markus Gerber, Marc Walter

**Affiliations:** ^1^Department of Sport, Exercise and Health, University of Basel, Basel, Switzerland; ^2^Deutsche Hochschule für Gesundheit und Sport GmbH, Ismaning, Germany; ^3^University Psychiatric Clinics, University of Basel, Basel, Switzerland

**Keywords:** exercise addiction, depression, ADHD, childhood trauma, exercise dependence

## Abstract

**Background:**

Exercise addiction is increasingly being recognized as a psychologically and physically burdensome set of symptoms. However, little is known about the psychiatric profiles of individuals who are at risk. It is well-established that individuals affected by substance use disorders frequently suffer from depression, attention deficit hyperactivity disorder (ADHD), and experiences of childhood trauma. The aim of this study is to determine whether this pattern of psychiatric disturbance is also present in individuals at risk for exercise addiction.

**Methods:**

Individuals exercising for 10+ h/week were divided into those at risk and not at risk for exercise addiction based on their scores on the Exercise Dependence Scale (EDS). Demographic data and scores on the Beck Depression Inventory (BDI), a measure for ADHD in adults and the Childhood Trauma Questionnaire (CTQ) were also gathered.

**Results:**

One hundred and twenty-three individuals agreed to participate in the study, and completed the questionnaires. Twenty-nine (23.6%) of these individuals were classed as at risk for exercise addiction. There was a statistically significant difference between the at-risk and not at-risk groups on the combined dependent variable after controlling for hours of exercise per week, *F*_(3, 95)_= 10.198, *p* = 0.00, Wilk's Λ = 0.756, partial η2 = 0.244. Compared to those not at risk, individuals at risk for exercise addiction had significantly higher scores for symptoms of depression [*t*_(121)_ = 4.944, *p* = 0.000], ADHD [*t*_(121)_ = 2.915, *p* = 0.004], and childhood trauma [*t*_(121)_ = 2.297, *p* = 0.024].

**Conclusion:**

Our results suggest that exercise addiction may be accompanied by a disturbed psychiatric profile consistent with other addictive disorders. Clinical interviewing in individuals at risk for exercise addiction is a valuable and worthwhile next step in characterizing this phenomenon.

## Introduction

Exercise addiction is a physically and psychologically burdensome phenomenon. First described in 1970 (Baekeland, 1970), addiction to exercise is reported to manifest as rigid adherence to exercise patterns, neglecting friends, family, and professional obligations for the sake of exercise, continuing despite illness, injury, and the recognition of these negative consequences, and severe withdrawal symptoms when planned exercise cannot be carried out (Freimuth et al., 2011). These symptoms reflect the characteristics of substance use disorders (Weinstein and Weinstein, 2014). However, exercise addiction, like other posited behavioral addictions, is not listed as a non-substance related disorder in the DSM 5 (Macfarlane et al., 2016). To date, studies addressing the phenomenon have provided insufficient evidence for the pathological nature of the symptoms (Potenza, 2014). In particular, the psychiatric profile of individuals who may be affected has received little attention.

Substance use and addictive disorders are frequently accompanied by other mental disorders. Various studies have reported on those which are most often diagnosed in treatment-seeking populations. A study involving 323 adults with various substance use disorders found a 74.3% prevalence of Axis I comorbidities, and 73.7% for Axis II disorders. The most common diagnoses were mood disorders, psychotic disorders, and anxiety disorders (Darcin et al., 2015). Another identified psychiatric comorbidities in 62.4% of a sample of alcohol dependent individuals (*n* = 101), with depressive and anxiety disorders being the most common (Gabriels et al., 2019). Depression is the most commonly diagnosed comorbidity in individuals with substance use disorders (Worley et al., 2012), with a 12-month prevalence of 32.8 and 44.3% in alcohol and drug use disorders, respectively (Grant et al., 2004).

Substance use disorders are characterized, physiologically, by disturbance of a number of neurotransmitter pathways associated with reward, including the dopaminergic and noradrenergic systems. Attention deficit hyperactivity disorder (ADHD) involves dysfunction of these systems, and as such, has been postulated as a disorder which may foster the development of substance use disorders (Ohlmeier et al., 2008). Attention deficit hyperactivity disorder, a disorder with a prevalence of about 5% in childhood, can persist into adulthood (Cortese and Coghill, 2018). Its primary symptoms are impaired concentration, poor response inhibition and impulse control, and excessive motor output (Hinshaw, 2018). It has been reported that ADHD presents a lifetime risk for substance use disorders twice as high as that of the general population; while some studies report a 25% ADHD prevalence in individuals who suffer from a substance use disorder (Dirks et al., 2017), others report estimates of between 35 and 75% prevalence in this population (Goodwin et al., 1975; Tarter et al., 1977).

A further factor often found in individuals with substance-related disorders is experience of traumatic events in childhood. Numerous studies indicate that exposure to childhood trauma is associated with substance-related disorders later in life (Nelson et al., 2006; Sinha, 2008). As with ADHD, childhood trauma, as a form of intense stress, also influences the dopaminergic system, meaning that individuals with higher stress reactivity, and exposure during early childhood, may be more sensitized to the rewarding effects of substances of abuse (Lijffijt et al., 2014). In one retrospective study of 8,613 individuals, each single traumatic childhood experience was found to increases the likelihood of early initiation to substance use between two- and four-fold, and individuals with five or more traumatic experiences were between seven and ten more likely to report problems with substances (Dube et al., 2003).

The evidence for the co-occurrence of behavioral addictions, with each other or with substance use disorder, is somewhat limited. A review of studies assessing mental disorders comorbid with problematic internet use found high prevalence rates of depression, ADHD, anxiety, and obsessive-compulsive symptoms (Carli et al., 2013). Similar findings for depression, video gaming, and gambling have also been reported (Yau et al., 2013). Na et al. (2017) reported that individuals suffering from comorbid internet and alcohol use disorders had more severe psychological symptoms, including higher levels of depression, than those suffering from only one of the two disorders (Na et al., 2017). Gambling disorder, the most widely researched behavioral addiction, has been reported to co-occur with psychological distress, buying and internet gaming addiction (Ford and Hakansson, 2020), binge eating disorder (Tang et al., 2020), anxiety and cognitive distortions (Barrault and Vareson, 2013), and depression (Martin et al., 2014). In a nationwide survey of households on the United States, individuals with gambling use disorder reported high rates of alcohol use disorders (73.2%), drug use disorders (38.1%), mood disorders (49.6%), and personality disorders (60.8%) (Petry et al., 2005).

In general, eating disorders, particularly anorexia nervosa and bulimia nervosa, are the mental disorders most commonly linked to exercise addiction (Trott et al., 2020). Indeed, it has been suggested that exercise addiction is only a secondary symptom of eating disorders, and should not be classified as a distinct disorder. While some scholars have suggested that exercise addiction is not a form of behavioral addiction, but a symptom which may be present in some eating disorders (Bamber et al., 2003), a number of studies point out that exercise addiction is reported in individuals who show no eating disorder symptoms (Griffiths, 1997; Grandi et al., 2011). Nevertheless, between 39 and 48% of individuals with eating disorders have been reported to be at risk for exercise addiction (Cook et al., 2013), and comorbidity of eating disorders and substance use disorders has been reported at between 17 and 46% (Harrop and Marlatt, 2010). In light of this persistent discussion, we suggest that much can be learned about the nature of exercise addiction if the prevalence of other mental disorders is also assessed. This data will indicate whether exercise addiction matches the profile of other substance use and addictive disorders, and shed light on whether a spectrum of mental disorders is present in this population.

A small number of studies have addressed other mental disorders in populations at risk of exercise addiction. While two studies found higher depression scores among at-risk individuals (Li et al., 2015; Weinstein et al., 2015), three others found no such link (Mayolas-Pi et al., 2017; Jee and Eun, 2018; Levit et al., 2018). Back et al. (2019) suggest that suffering from anxiety is a risk factor for symptoms of exercise addiction (Back et al., 2019). Individuals with ADHD are also more likely to exercise excessively, compared to those without (Berger et al., 2014).

In summary, individuals who suffer from substance use disorders also frequently suffer from depression and/or ADHD, and have frequently experienced childhood trauma. This disturbed psychiatric profile is common across all forms of substance use disorder, and appears to be present in non-substance related disorders as well. Comorbid mental disorders may contribute to the development of addictive disorders, be maintained by them, and/or develop as a result of them (Raskin and Miller, 1993). In substance use disorders, there is a broad range of evidence regarding the ways in which exposure to drugs can influence neurobiological development, and conversely, the psychiatric characteristics which may predict substance abuse (Volkow, 2004). In the field of behavioral addictions, however, this detailed psychological assessment is still in the early stages. Furthermore, given the persisting discussions regarding the clinical classification of many behavioral addictions, including exercise addiction, more thorough examination of the psychiatric profiles of affected individuals is currently lacking. It might, for instance, shed more light on whether these potential disorders are more “compulsive” or “addictive” in nature (Grant et al., 2004). It may also indicate whether certain psychological disorders tend to predispose individuals toward particular behaviors which then function as maladaptive coping mechanisms (Starcevic and Khazaal, 2017).

The aim of this study is to determine whether individuals classified as at risk for exercise addiction have higher scores of depression, ADHD, and childhood trauma, compared to individuals not at risk who nonetheless exercise frequently. We hypothesize that this will be the case for all three parameters. Furthermore, we will screen for diagnoses of eating disorders, depression, ADHD, anxiety disorders, and other mental disorders, in order to provide further evidence on disorders which appear to co-occur with exercise addiction. This study will contribute important information on the psychiatric profiles of individuals at risk for exercise addiction, and serve to provide evidence for characterizing the phenomenon.

## Methods

### Participants

In order to identify individuals likely to be at risk of exercise addiction, specific inclusion and exclusion criteria were formulated for this study. It was not our aim to assess prevalence in the general population; rather, we aimed to recruit as many individuals as possible at risk of exercise addiction. This was due to the fact that we aimed to achieve adequate power for subsequent phases of this study. However, we anticipated that we would recruit many frequent exercisers who turned out not to be at risk for exercise addiction, based on the more conservative prevalence estimates in exercising populations (Breuer and Kleinert, 2009), and consequently, that the group sizes of those at risk vs. not at risk for exercise addiction were likely to be unequal. This data is valuable because it enables a comparison of the psychiatric profiles of those who report being troubled by their exercise habits, and those who merely exercise frequently. Hence, the following criteria were employed:

Inclusion criteria; (a) 10 or more hours of exercise per week, (b) continuing to exercise despite illness or injury, (c) aged between 18 and 70 years, and (d) fluency in German. Exclusion criteria; (a) regular weeks with fewer than 10 h of exercise, (b) exercising only with a mild cold, but stopping exercise for anything more severe.

From June 2019, participants were recruited via online advertising on the University of Basel public forum, the forums of various exercise clubs and interest groups, via flyers in fitness centers, sports clubs, and sporting venues, posters on public transportation and outdoor poster boards, and the website www.bewegungssucht.ch. Participants were informed that they would receive 40 CHF for completing the initial phase of the study.

Participants contacted the study team via email or telephone call. A set of questions was asked, in order to confirm that inclusion criteria were met, and exclusion criteria were absent, and if eligible, participants were invited to read and sign the informed study consent, and complete the first phase of the study. Two subsequent phases, a clinical interview and FMRI, are ongoing and will be reported in subsequent publications. Prior to the outbreak of COVID-19, an investigator met participants and provided the questionnaires in paper form. Following the implementation of social distancing measures, questionnaires were completed in electronic form.

### Measures

Participants reported their age, highest level of education, employment, and relationship status. They provided details about the amount and type of exercise they engage in, whether they participated in competitions, and whether they had previously been diagnosed with an eating disorder, ADHD, depression, anxiety, or any other mental disorder.

#### Exercise Dependence Scale

The Exercise Dependence Scale (EDS) is a 21-item questionnaire assessing symptoms of exercise addiction. The questionnaire includes seven subscales (tolerance, withdrawal, intention effects, lack of control, time, reduction in other activities, and continuance), each of which is assessed by three questions graded on a six-point Likert-type scale. A score of 15 or more on at least three of the seven subscales indicates the respondent is at risk; a score of between 9 and 14 on at least three subscales indicates the respondent is symptomatic but not at risk; scores below this are classed as non-symptomatic. The German translation of the EDS has been shown to have good psychometric properties (Müller et al., 2014). The Cronbach's alpha for all 21 items in the present sample α = 0.83. In this study, we use the threshold of “at risk” to categorize individuals who are or are not potentially addicted to exercise. An example of an item is: “I end up exercising for longer than I planned.”

#### Childhood Trauma Questionnaire

The Childhood Trauma Questionnaire (CTQ) is a 34 item instruments assessing traumatic experiences in six domains (death, divorce, violence, sexual abuse, illness, or other) during early childhood. Answers are given on a five-point Likert-type scale, with the lowest scores indicating no to low trauma, and the highest indicating severe to extreme trauma. A higher sum score indicates greater trauma. The German translation shows good reliability and validity (Wingenfeld et al., 2010). For this sample, the Cronbach's alpha was α = 0.87. An example of an item is: “As a child, I was struck so hard by a member of my family that I had to be taken to a doctor/the hospital.”

#### Homburger ADHS Skalen für Erwachsene (Homburger ADHD Scale for Adults)

The Homburger ADHS Skalen für Erwachsene (HASE) is used to investigate symptoms of ADHD in adults. The instrument contains 22 items which are self-rated on a four-point Likert-type scale; higher scores indicate more severe symptoms. The psychometric properties of the HASE are satisfactory (Schmidt and Petermann, 2018). Internal consistency for the current sample was α = 0.70. An example of an item is: “I find it difficult to sit still for long periods of time (e.g., at the theater or cinema):”

#### Beck Depression Inventory

The Beck Depression Inventory (BDI) (Beck et al., 1961) is used to assess the severity of depressive symptoms. The BDI consists of 21 items including a range of affective, behavioral, cognitive, and somatic symptoms that are indicative of unipolar depression. Respondents are asked to select from four responses that reflect increasing levels of depressive symptomatology. Possible sum scores range from 0 to 63 with higher scores indicating more depressive symptoms (cut-off for mild-to-moderate depression ≥10). Adequate validity and reliability of the BDI has been shown previously (Beck et al., 1988). For the current sample, Cronbach's alpha was α = 0.78. An example of an item (denoting the most severe symptoms) is: “I blame myself for everything when things go wrong:”

### Statistical Analysis

Data were analyzed using IBM SPSS Statistics 26. For demographic variables, we calculated means and standard deviations, sum scores, and employed independent sample *T*-tests and Chi Squared tests to determine whether there were differences between groups. For the main outcome variables, we employed a multivariate analysis of covariance (MANCOVA), controlling for the number of exercise hours engaged in per week. These were further explored with independent sample *T*-tests. The threshold for significance was set at *p* = 0.05. Due to the difference in group sizes, we calculated Levene's test of homogeneity of variances and robust tests of equality of means (Welch and Brown-Forsythe tests).

### Ethics

The study procedures were carried out in accordance with the Declaration of Helsinki. The ethical review board of Northwestern Switzerland reviewed and approved the study.

## Results

One hundred and sixty-one individuals contacted the investigators upon learning about the study. Twenty-eight did not meet the inclusion criteria, and four agreed to participate in the study but failed to complete the questionnaires. One hundred and twenty-nine individuals agreed to participate in the study, and completed the questionnaires; six failed to complete several measures, so the final sample is based on 123 completed questionnaires. Twenty-nine of these individuals were classed as at risk for exercise addiction, based on their EDS responses. Table 1 shows demographic data for the sample. Individuals at risk for exercise dependence exercised for significantly more hours per week, and were significantly more likely to have a previous diagnosis of an anxiety disorder; otherwise, there were no differences between groups.

**Table 1:** Descriptive and inferential statistics for demographic data.

	**At risk for exercise addiction** **(*n* = 29)**	**Not at risk for exercise addiction** **(*n* = 94)**		
	***M (SD)***	***M (SD)***	***t***	***p***
Age	31.55 (15.30)	33.29 (14.24)	−0.564	0.574
Hours of exercise per week	15.07 (8.59)	11.57 (3.43)	3.216	0.002^***^
	***n***	***n***	***χ^2^***	***P***
Gender				
Male	15	64	2.830	0.120
Female	14	29		
Relationship status				
Single	17	58	1.950	0.377
Married	4	18		
Divorced	2	2		
Educational level				
High school	6	16	2.718	0.257
Diploma	12	31		
University	5	32		
Previous psychiatric diagnoses				
Eating disorder	4	4	3.315	0.069
ADHD	1	1	0.788	0.375
Depression	2	6	0.010	0.922
Anxiety	3	1	6.068	0.014^*^
Other	1	2	0.162	0.687

* *p < 0.05,*

*** *p < 0.001*.

Levene's test of homogeneity of variances was significant for group differences on the BDI and EDS scores. However, using the more robust Welch's and Brown-Forsythe tests for equality of means, the group differences for these variables remained highly significant. There was a statistically significant difference between the at-risk and not at-risk groups on the combined dependent variable after controlling for hours of exercise per week, *F*_(3, 95)_ = 10.198, *p* < 0.001, Wilk's Λ = 0.756, partial η^2^ = 0.244. Specifically, those above the cut-off for exercise addiction had significantly to highly significantly greater scores on the BDI, CTQ, and HASE, indicating more symptoms of each of these disorders. Table 2 displays the scores on the questionnaires, and inferential statistics, separately for both groups.

**Table 2:** Inferential statistics for main outcome variables.

	**At risk for exercise addiction** **(*n* = 29)**	**Not at risk for exercise addiction** **(*n* = 94)**		
	***M (SD)***	***M(SD)***	***t***	***p***
BDI	12.55 (9.90)	5.77 (4.98)	4.944	0.000^***^
HASE	19.93 (14.27)	13.46 (8.99)	2.915	0.004^***^
CTQ	52.17 (18.16)	44.90 (11.57)	2.297	0.024^*^

*BDI, Beck Depression Inventory; HASE, Homburger ADHS Skalen für Erwachsene; CTQ, Childhood Trauma Questionnaire.*

* *p < 0.05,*

*** *p < 0.001*.

## Discussion

Our study shows that individuals at risk of exercise addiction suffer from symptoms of mental disorders to a greater degree than regular exercisers who are not at risk. Scores on self-report measures for depression, ADHD, and childhood trauma were significantly higher in the at-risk group. Disorders in these domains are common in substance use and other addictive disorders. Below, we discuss the implications of these findings in detail.

### Psychiatric Profile Similar to Substance Use Disorders

Our results show that in three psychiatric domains which are commonly disturbed in individuals with substance use disorders, those at risk for exercise addiction also report disturbance. A fairly high exercise volume (10 + h per week) itself does not, therefore, appear to be accompanied by psychiatric disturbance. Rather, the particular symptoms of exercise addiction are linked to disturbance in other domains. Depression, the most commonly diagnosed disorder amongst individuals with substance use disorders, was highly significantly more frequent at-risk participants in our sample. This is also true for symptoms of ADHD; for childhood trauma, differences also reach significance. These results confirm the findings of other studies which have found higher scores on mental disorder tests in those at risk for exercise addiction (Li et al., 2015; Weinstein et al., 2015; Back et al., 2019). This is also the first time that childhood trauma has been investigated in the context of exercise addiction, and our results indicate that this is a field which has clear relevance for future investigations and treatment approaches.

A key element of the argument urging caution against pathologizing behaviors is the fact that, unlike illegal drug consumption, the behaviors in question are typically a part of a healthy life (Billieux et al., 2015). It would be undesirable to consider a relatively high volume of a normal behavior as unhealthy, or indeed pathological. However, if there is evidence that the behavior is pursued despite psychiatric distress, the argument for defining it as a disorder becomes more compelling. Our study indicates that there is a clear separation amongst individuals with a high exercise volume; those who report suffering due to their exercise habits also have a disturbed psychiatric profile. This is evidence to indicate that excessive exercise is troubling on a psychiatric level, and warrants clinical investigation for potential DSM categorization.

### Diagnoses of Eating Disorders No More Common in At-Risk Group

A topic of debate in the literature is the degree to which exercise addiction can be disentangled from disordered eating (Cunningham et al., 2016). The majority of studies which address comorbidities in exercise addiction focus on eating disorders. As noted above, this is because many individuals diagnosed with eating disorders exercise excessively as method of purging calories or accelerating weight loss (Cook et al., 2013). A focus on populations with eating disorders a s possible risk group for exercise addiction is not unwarranted; nevertheless, if the two disorders are distinct from one another, continued focus on eating habits may be unhelpful to fully understanding the causes and perpetuating factors of exercise addiction in some individuals. Our study indicates that four of those at risk for exercise addiction had a diagnosis of an eating disorder; this rate does not differ from the diagnoses in those not at-risk, although the difference is approaching significance. While we did not screen for eating disorder symptoms, we suggest that this finding, combined with the data on psychiatric disturbance, suggests that factors other than eating disorders co-occur with exercise addiction. Other studies have reported high scores on measures of exercise addiction in the absence of eating disorder symptoms of diagnoses (Grandi et al., 2011). We therefore suggest that, while it is certainly the case that many sufferers of eating disorders exercise excessively, there is no robust evidence that exercise addiction is solely a symptom of disordered eating.

### Psychiatric History and Clinical Diagnoses Are Required

Our results indicate that symptoms of psychiatric disturbance are present in those at risk of exercise addiction. However, clinical diagnoses were only made in a small group of our participants. Based on previous diagnoses, the only group difference found was for anxiety disorders, which had been diagnosed more frequently in the at-risk group. Interestingly, although scores for symptoms of depression were higher in the at-risk group, there was no difference between the groups regarding a previous diagnosis of depression. It is to be expected that many participants, in both groups, will never have consulted a psychiatrist; consequently, our findings regarding previous diagnoses must be interpreted with caution.

Our cross-sectional data do not permit us to speculate about whether symptoms of depression and ADHD predate those of exercise addiction, though childhood trauma, by default, is highly likely to have emerged first. Clinical interviewing will shed more light on whether depression, and possibly ADHD, precede, co-occur with, or result from the onset of exercise dependence. This information will contribute toward the development of treatment programs specifically addressing exercise addiction.

### Practical Implications and Recommendations for Future Research

The main key implication of these findings is that, in cases of a suspected or likely addiction to exercise, practitioners, and therapists should consider screening for other psychiatric disorders. These may underlie the exercise symptoms, and therefore should not be ignored in a treatment plan. Secondly, we suggest that there is no call to be dubious about diagnosis of excessive exercise habits in the absence of disordered waiting behavior or body image issues; physicians may specifically address these behaviors in the context of a psychiatric disturbance. We would urge practitioners who suspect excessive exercise behavior to use one of the screening questionnaires currently available, and discuss the results with patients in terms of their mental well-being.

Future research should expand on the psychiatric profile of individuals who exercise to excess. These exercise behaviors, and the potential negative burdensome effects, must also be systematically cataloged, in order to advance exercise addiction from a suspected to a confirmed diagnosis.

## Conclusion

Our study indicates that individuals at risk for exercise addiction have higher scores for symptoms of depression, ADHD, and childhood trauma. These disorders are all frequently present in individuals suffering from substance use disorders. Our results indicate that exercise addiction may be accompanied by a disturbed psychiatric profile consistent with addictive disorders. Clinical interviewing in individuals at risk for exercise addiction is a valuable and worthwhile next step in characterizing this phenomenon.

## Data Availability Statement

The raw data supporting the conclusions of this article will be made available by the authors, without undue reservation.

## Ethics Statement

The studies involving human participants were reviewed and approved by Ethikkomission Nordwestschweiz. The patients/participants provided their written informed consent to participate in this study.

## Author Contributions

FC conceived of the study, carried out the investigation, wrote, and revised the paper. UB, AS, GW, UL, UP, and MG revised the paper. MW conceived of the study and revised the paper. All authors contributed to the article and approved the submitted version.

## Funding

This work was funded by the Gertrud Thalmann Fonds.

## Conflict of Interest

Author UB was employed by Deutsche Hochschule für Gesundheit und Sport GmbH. The remaining authors declare that the research was conducted in the absence of any commercial or financial relationships that could be construed as a potential conflict of interest.

## Publisher's Note

All claims expressed in this article are solely those of the authors and do not necessarily represent those of their affiliated organizations, or those of the publisher, the editors and the reviewers. Any product that may be evaluated in this article, or claim that may be made by its manufacturer, is not guaranteed or endorsed by the publisher.
